# Weakly acidic carboxy group-grafted β-cyclodextrin-threaded acid-degradable polyrotaxanes for modulating protein interaction and cellular internalization

**DOI:** 10.1080/14686996.2021.1935315

**Published:** 2021-06-29

**Authors:** Shunyao Zhang, Atsushi Tamura, Nobuhiko Yui

**Affiliations:** Department of Organic Biomaterials, Institute of Biomaterials and Bioengineering, Tokyo Medical and Dental University (TMDU), Tokyo, Japan

**Keywords:** Polyrotaxane, β-cyclodextrin, carboxy group, protein interaction, intracellular uptake, 30 Bio-inspired and biomedical materials, 301 Chemical syntheses / processing, 101 Self-assembly / Self-organized materials, 211 Scaffold / Tissue engineering/Drug delivery

## Abstract

To improve the therapeutic potential of β-cyclodextrin (β-CD)-threaded acid-degradable polyrotaxanes (β-CD PRXs) in cholesterol-related metabolic disorders, we investigated the effect of carboxylation of β-CD PRXs on intracellular uptake. In this study, we established a synthetic method for the modification of carboxylalkyl carbamates on β-CD PRXs without degradation and synthesized three series of carboxyalkyl carbamate group-modified β-CD PRXs with different alkyl spacer lengths. The modification of carboxymethyl carbamate (CMC), carboxyethyl carbamate (CEC), and carboxypropyl carbamate (CPC) on the β-CD PRXs slightly reduced the interaction of the PRXs with the lipid layer model compared with the modification of 2-(2-hydroxyethoxy)ethyl carbamate (HEE-PRX), which was used in our previous studies. However, all the carboxylated β-CD PRXs showed a significantly stronger interaction with a protein model compared with HEE-PRX. The carboxylated β-CD PRXs showed significantly high intracellular uptake, through macrophage scavenger receptor A (MSR-A)-mediated endocytosis, in MSR-A-positive RAW 264.7 cells compared with HEE-PRX. Interestingly, the carboxylated β-CD PRXs also showed significantly higher intracellular uptake even in MSR-A-negative cells compared with HEE-PRX. Carboxylated β-CD PRXs are considered to strongly interact with other membrane proteins, resulting in high intracellular uptake. The length of the alkyl spacer affected the intracellular uptake levels of carboxylated PRXs, however, this relationship was varied for different cell types. Furthermore, none of the carboxylated β-CD PRXs exhibited cytotoxicity in the RAW 264.7 and NIH/3T3 cells. Altogether, carboxylation of β-CD PRXs is a promising chemical modification approach for their therapeutic application because carboxylated β-CD PRXs exhibit high cellular internalization efficiency in MSR-A-negative cells and negligible toxicity.

## GRAPHICAL ABSTRACT

**Figure:**
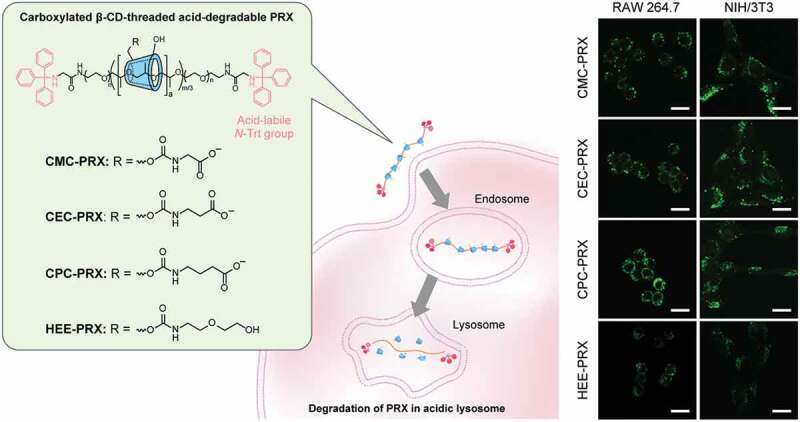

## 1. Introduction

Cyclodextrin (CD)-threaded polyrotaxane (PRX) is a representative supramolecular polymer, in which several CDs are threaded along a linear polymer chain and interlocked onto the polymer axis through capping with bulky stopper molecules [1–3]. PRXs have recently received considerable attention as a new class of polymeric materials because the molecular mobility of threaded cyclic molecules imparts new material functions [4]. Another advantage of PRXs for material applications is their stimuli-responsive dissociative character [5,6]. When stimuli-cleavable chemical bonds are introduced in the axle polymer, the resulting PRXs readily dissociate into their constituent molecules in response to various chemical and physical stimuli, such as pH, light, reductive molecules, and reactive oxygen species (ROS) [7–10]. These stimuli-degradable PRXs have been applied as delivery carriers of drugs, therapeutic genes, and proteins for achieving immediate release of therapeutic payloads through the stimuli-induced degradation of the PRXs in intracellular environments (*e.g*. acidic pH and reductive glutathione) [9–15].

**Figure 1.**
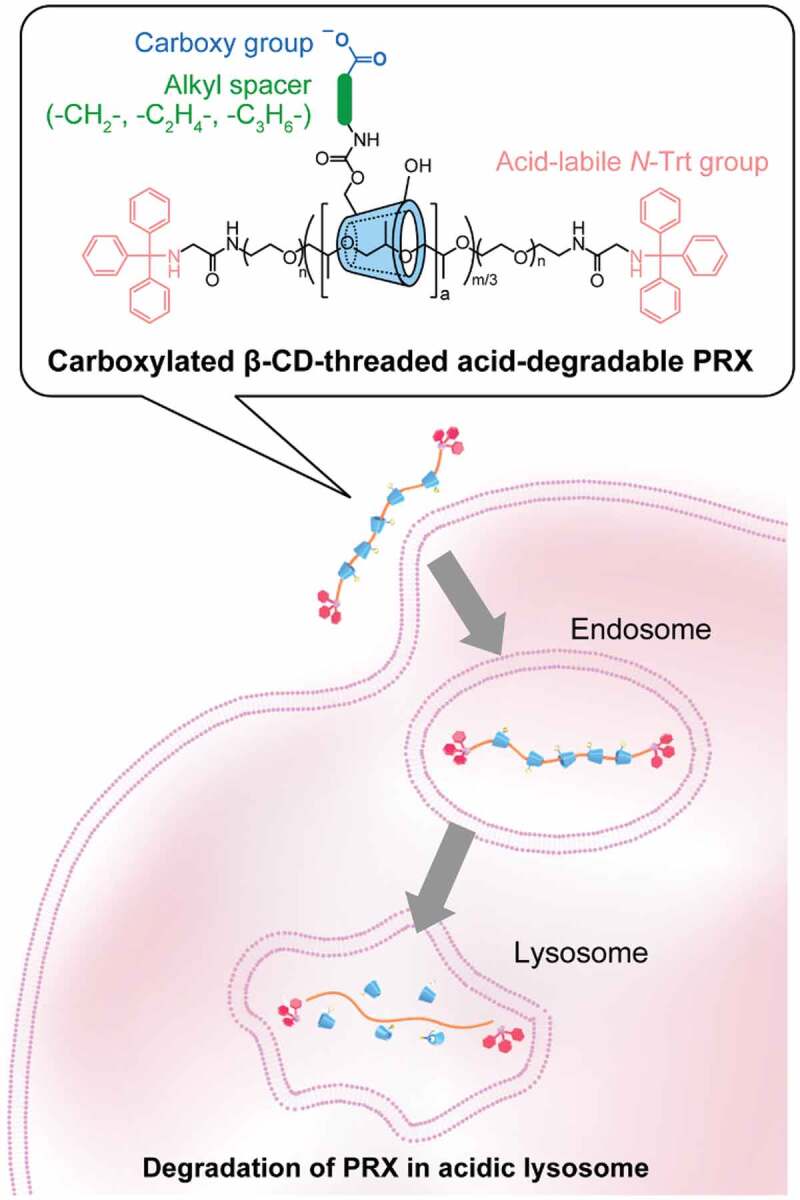
Schematic illustration for the cellular internalization and intracellular degradation of carboxylated β-cyclodextrin (β-CD)-threaded acid-degradable polyrotaxanes (PRXs) with different alkyl spacer lengths

Taking advantage of the stimuli-degradable character of PRXs, our research groups have proposed the therapeutic application of PRXs without conjugation and complexation of therapeutic drugs [7,16–18]. Recently, β-CD derivatives have demonstrated therapeutic effects in several intractable diseases such as Niemann-Pick type C (NPC) disease, atherosclerosis, diabetic kidney diseases, and Alport syndrome [19–23], most likely through the encapsulation of cellular cholesterol in its hydrophobic cavity. In particular, clinical trials are currently ongoing for 2-hydroxypropyl β-CD (HP-β-CD) in NPC disease, which is an autosomal recessive lysosomal trafficking disorder that exhibits abnormal cholesterol accumulation in lysosomes [24]. However, phase I/IIa clinical trials revealed that intrathecal administration of HP-β-CD induced audiotoxicity [24], and the safety of β-CD derivatives is controversial. The toxic effects of β-CD derivatives arise from the extraction of cholesterol from the plasma membrane [25,26]. In contrast, we observed that the toxic effects of β-CDs can be masked by their incorporation into PRXs [16]. Additionally, for the treatment of NPC disease, we designed β-CD-threaded acid-degradable PRXs (β-CD PRXs) that can release threaded β-CDs in lysosomes through the acid-induced degradation of PRXs (Figure 1). Acid-degradable β-CD PRXs have been demonstrated to inhibit cholesterol accumulation and prolong survival at a lower dose than HP-β-CD in a mouse model of NPC disease [17]. Therefore, the acid-degradable β-CD PRXs are promising candidates for both attenuating toxicity and potentiating the therapeutic efficacy of β-CD in NPC disease.

In our previous studies, acid-degradable β-CD PRXs were chemically modified with hydrophilic oligo(ethylene glycol) chains (*e.g*. 2-(2-hydroxyethoxy)ethyl carbamate; HEE) for increasing the solubility in aqueous media [7,17]. Although the chemical modifications of PRXs are essential for improving their solubility in aqueous media, the highly hydrophilic functional groups generally disturb cellular internalization and tissue accumulation. To further improve the therapeutic efficacy of β-CD PRXs, the molecular design of PRXs should be optimized. One potential method for improving the cellular internalization efficiency of PRXs is modification with targeting biomolecules, such as peptides, saccharides, antibodies, and aptamers [27–31]. These targeting molecules interact with specific receptors, enabling selective and highly efficient targeting to specific cells. However, chemical modification of these targeting biomolecules generally requires delicate reaction conditions, and it is difficult to achieve scalable synthesis. Another potential approach for improving the cellular internalization efficiency of PRXs is optimization of the physicochemical characters of PRXs, such as hydrophilicity–hydrophobicity, charge, molecular weight, and threading ratio of CDs. Among these, the chemical modification of charged functional groups potentially improves cellular internalization efficiency [32,33], because both cationic and anionic functional groups facilitate the interaction of PRXs with proteins through electrostatic interactions. We previously reported that weakly acidic carboxymethyl ether-modified α-CD PRX showed selective and high intracellular uptake in a macrophage-like cell line (RAW 264.7 cells), whereas it exhibited negligible uptake in fibroblasts (NIH/3T3 cells) [34]. We considered that the carboxy groups increased the interaction with macrophage scavenger receptor A (MSR-A), which recognizes anionic macromolecules [35], resulting in selective and high intracellular uptake in macrophage-like cell lines. Because macrophages plays a central role in the progression of atherosclerosis and inflammatory diseases [36,37], carboxylation of acid-degradable β-CD PRXs is promising for therapeutic applications in cholesterol-related metabolic diseases such as atherosclerosis.

However, previous methods to modify weakly acidic carboxy groups cannot be applied to acid-degradable β-CD PRXs, because acidic carboxy groups lead to the degradation during the reaction and purification processes. In this study, we developed a new synthetic method for the carboxylation of acid-degradable PRX using protected carboxy ester derivatives. This method allows us to modify various carboxy groups without degrading the acid-degradable β-CD PRXs. We synthesized three series of carboxylated β-CD PRXs with different alkyl spacer lengths between the carboxy group and threaded β-CD and investigated their cellular internalization efficiency (Figure 1). A unique observation from our study is that the carboxylated β-CD PRXs showed high cellular internalization efficiency, regardless of the expression of MSR-A. We expected that carboxylation of acid-degradable β-CD PRXs would be a promising design for improving the therapeutic efficacy of PRXs.

## 2. Materials and methods

### 2.1. Materials

As described in **Supplementary text**, we synthesized acid-degradable PRX, composed of β-CD as a cyclic molecule, PEG-*b*-PPG-*b*-PEG (Pluronic P123) as an axial polymer, and *N*-triphenylmethyl groups as acid-labile stopper molecule. In this study, acid-degradable β-CD PRX with 12.5 threaded β-CDs and a number-average molecular weight of 20,500 was utilized for the synthesis of carboxylated PRXs. HEE-PRX was synthesized as described in our previous study [7]. 1,1ʹ-Carbonyldiimidazole (CDI) was obtained from Merck (Darmstadt, Germany). Glycine ethyl ester hydrochloride, methyl 3-aminopropionate hydrochloride, and methyl 4-aminobutyrate hydrochloride were obtained from Tokyo Chemical Industry (Tokyo, Japan). Dehydrated dimethyl sulfoxide (DMSO) and triethylamine (TEA) were obtained from Fujifilm Wako Pure Chemical (Osaka, Japan). Other reagents and solvents were obtained from Fujifilm Wako Pure Chemical and Kanto Chemical (Tokyo, Japan)

### 2.2. Instrumentation

^1^H nuclear magnetic resonance (NMR) spectra were recorded using a Bruker Avance III 400 MHz spectrometer (Bruker BioSpin, Rheinstetten, Germany) in DMSO-*d*_6_ and D_2_O at 25°C. Chemical shifts in ^1^H NMR spectra were referenced using DMSO (2.5 ppm in DMSO-*d*_6_) and HDO (4.65 ppm in D_2_O). Size exclusion chromatography (SEC) was performed using a Prominence-i LC-2030 Plus system (Shimadzu, Kyoto, Japan) equipped with an RID-20A refractive index detector and a combination of TSKgel α-4000 and α-2500 columns (300 mm length, 7.8 mm internal diameter; Tosoh, Tokyo, Japan). Sample solutions were injected into the system and eluted with DMSO containing 10 mM LiBr at a flow rate of 0.35 mL/min at 60°C. Fourier-transform infrared (FT-IR) spectra were recorded using a Spectrum 100 FTIR spectrometer (Perkin Elmer, Wellesley, MA, USA) equipped with an HgCdTe (MCT) detector. The sample powders were ground with KBr for preparing pellets for the analysis.

### 2.3. Carboxylation of acid-degradable PRXs

**Figure 2.**
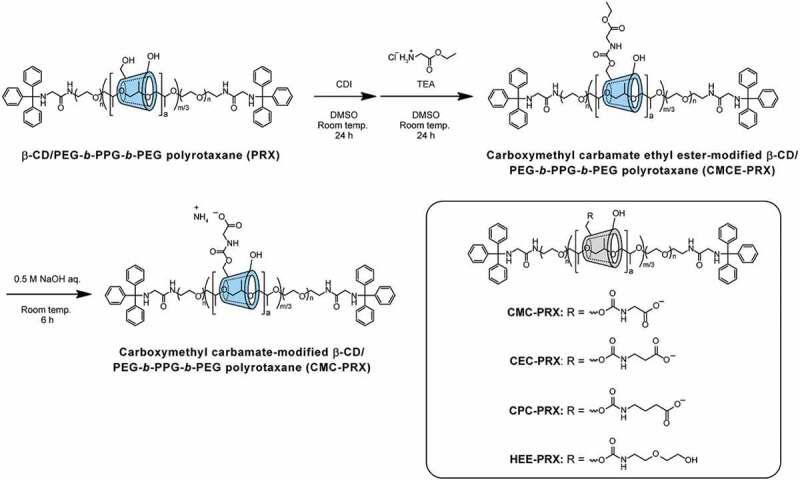
Scheme for the synthesis of carboxymethyl carbamate (CMC)-modified β-CD PRX, and the chemical structures of CMC-PRX, 2-carboxyethyl carbamate-modified β-CD PRX (CEC-PRX), 3-carboxypropyl carbamate-modified β-CD PRX (CPC-PRX), and 2-(2-hydroxyethoxy)ethyl carbamate-modified β-CD PRX (HEE-PRX)

Three series of carboxylated acid-degradable PRXs were synthesized, as shown in Figure 2(a). The synthesis of carboxymethyl carbamate-modified PRX (CMC-PRX) was as follows: Briefly, PRX (200 mg, 9.76 μmol PRX, and 122 μmol threaded β-CD) and CDI (140 mg, 863 μmol) were dissolved in dehydrated DMSO (10 mL), and the solution was stirred for 24 h at room temperature under a nitrogen atmosphere. Glycine ethyl ester hydrochloride (1.2 g, 8.6 mmol) and TEA (1.2 mL, 8.59 mmol) dissolved in dehydrated DMSO (5 mL) were then added into the mixed solution, and the solution was stirred for additional 24 h at the room temperature. After the reaction, the solution was purified by dialysis against deionized water using a Spectra/Por 1 dialysis membrane (molecular weight cut-off of 6000–8000; Spectrum Laboratories, CA, USA) for 3 days at 4°C. Subsequently, the solution was freeze-dried to yield carboxymethyl carbamate ethyl ester-modified PRX (CMCE-PRX; 240 mg, 98.1% yield). ^1^H NMR (400 MHz, DMSO-*d*_6_): δ (ppm) = 1.03 (m, – C***H_3_*** of Pluronic P123), 1.18 (m, – O-CH_2_-C***H_3_***), 3.0–4.0 (m, – NH-C***H_2_***-C(=O) – of CPC group, – C***H_2_***C***H_2_***O – and – C***H_2_***-C***H*** – of PEG and PPG segments in Pluronic P123, respectively, and H2, H3, H4, H5, and H6 protons of β-CD), 4.10 (m, – O-C***H_2_***-CH_3_), 4.35–4.65 ppm (m, O_6_H proton of β-CD), 4.7–5.2 (m, H_1_ proton of β-CD), 5.4–6.0 ppm (m, O_2_H and O_3_H protons of β-CD), 7.1–7.7 (m, – N***H***-CH_2_-C(=O) – of CMCE group and Trt group).

For the deprotection of carboxy ethyl esters, CMCE-PRX (240 mg) was dissolved in 0.5 M NaOH (10 mL) and allowed to react for 6 h at room temperature. After neutralization with HCl, the solution was dialyzed against 0.1% ammonia solution for 2 days at 4°C and deionized water for 1 day at 4°C using Spectra/Por 1. Subsequently, the solution was freeze-dried to yield CMC-PRX as an ammonium salt (150 mg, 63.4% yield). ^1^H NMR (400 MHz, D_2_O): δ (ppm) = 1.12 (m, – C***H_3_*** of Pluronic P123), 3.2–4.5 (m, – NH-C***H_2_***-C(=O) – of CMC group, – C***H_2_***C***H_2_***O – and – C***H_2_***-C***H*** – of PEG and PPG segments in Pluronic P123, respectively, and H2, H3, H4, H5, and H6 protons of β-CD), 4.9–5.3 (m, H_1_ proton of β-CD), 7.1–7.5 (Trt group).

Carboxylethyl carbamate methyl ester-modified PRX (CECM-PRX) was synthesized using methyl 3-aminopropionate hydrochloride in the same manner as CMCE-PRX (224 mg, 95.5% yield). ^1^H NMR (400 MHz, DMSO-*d*_6_): δ (ppm) = 1.03 (m, – C***H_3_*** of Pluronic P123), 2.45 (m, – NH-CH_2_-C***H_2_***-C(=O) – of CECM group), 3.19 (m, – NH-C***H_2_***-CH_2_-C(=O) – of CECM group), 3.25–4.27 (m, – C***H_2_***C***H_2_***O – and – C***H_2_***-C***H*** – of PEG and PPG segments in Pluronic P123, respectively, and H2, H3, H4, H5, and H6 protons of β-CD, and – C(=O)-O-C***H_3_*** of CECM group), 4.27–4.7 ppm (m, O_6_H proton of β-CD), 4.7–5.2 (m, H_1_ proton of β-CD), 5.4–6.1 ppm (m, O_2_H and O_3_H protons of β-CD), 6.7–7.45 (m, – N***H***-CH_2_-C(=O) – of CECM group and Trt group).

The deprotection of methyl ester was performed in 0.5 M NaOH for 6 h, which yielded carboxylethyl carbamate-modified PRX as an ammonium salt (CEC-PRX; 146 mg, 65.0% yield). ^1^H NMR (400 MHz, D_2_O): δ (ppm) = 1.15 (m, – C***H_3_*** of Pluronic P123), 2.32 (m, – NH-CH_2_-C***H_2_***-C(=O) – of CEC group), 3.25 ‘(m, – NH-C***H_2_***-CH_2_-C(=O) – of CEC group), 3.25–4.55 (m, – C***H_2_***C***H_2_***O – and – C***H_2_***-C***H*** – of PEG and PPG segments in Pluronic P123, respectively, and H2, H3, H4, H5, and H6 protons of β-CD), 4.85–5.2 (m, H_1_ proton of β-CD), 7.15–7.45 (Trt group).

Carboxylpropyl carbamate methyl ester-modified PRX (CPCM-PRX) was synthesized using methyl 4-aminobutyrate hydrochloride in the same manner as CMCE-PRX (218 mg, 94.2% yield). ^1^H NMR (400 MHz, DMSO-*d*_6_): δ (ppm) = 1.03 (m, – C***H_3_*** of Pluronic P123), 1.64 (m, – NH-CH_2_-C***H_2_***-CH_2_-C(=O) – of CPCM group), 2.45 (m, – NH-CH_2_-CH_2_-C***H_2_***-C(=O) – of CPCM group), 2.94 (m, – NH-C***H_2_***-CH_2_-CH_2_-C(=O) – of CPCM group), 3.1–4.27 (m, – C***H_2_***C***H_2_***O – and – C***H_2_***-C***H*** – of PEG and PPG segments in Pluronic P123, respectively, and H2, H3, H4, H5, and H6 protons of β-CD, and – C(=O)-O-C***H_3_*** of CPCM group), 4.25–4.7 ppm (m, O_6_H proton of β-CD), 4.7–5.2 (m, H_1_ proton of β-CD), 5.4–6.1 ppm (m, O_2_H and O_3_H protons of β-CD), 6.7–7.45 (m, – N***H***-CH_2_-C(=O) – of CPCM group and Trt group).

The deprotection of methyl ester was performed in 0.5 M NaOH for 6 h to yield carboxylpropyl carbamate-modified PRX as an ammonium salt (CPC-PRX; 163 mg, 74.6% yield). ^1^H NMR (400 MHz, D_2_O): δ (ppm) = 1.13 (m, – C***H_3_*** of Pluronic P123), 1.66 (-NH-CH_2_-C***H_2_***-CH_2_-C(=O) – of CPC group), 2.12 (-NH-CH_2_-CH_2_-C***H_2_***-C(=O) – of CPC group), 3.04 (-NH-C***H_2_***-CH_2_-CH_2_-C(=O) – of CPC group), 3.1–4.55 (m, – NH-C***H_2_***-C(=O) – of CPC group, – C***H_2_***C***H_2_***O – and – C***H_2_***-C***H*** – of PEG and PPG segments in Pluronic P123, respectively, and H2, H3, H4, H5, and H6 protons of β-CD), 4.85–5.3 (m, H_1_ proton of β-CD), 7.15–7.45 (Trt group).

### 2.4. Potentiometric titration

Carboxylated PRXs and NaCl (11.7 mg) were dissolved in 10 mM NaOH (20 mL; NaCl concentration: 10 mM, carboxy group concentration: 5 mM). The solution was titrated with 10 mM HCl containing 10 mM NaCl at 25°C using an AUT-701 automatic titrator (DKK-TOA, Tokyo, Japan). The titrant was added in quantities of 40 μL at intervals of 10 s. The p*K*_a_ value and degree of ionization were calculated from the pH–α curves.

### 2.5. Transmittance measurement

The carboxylated PRXs were dissolved in phosphate buffer (NaH_2_PO_4_/Na_2_HPO_4_, pH 6–8), acetate buffer (CH_3_COOH/CH_3_COONa, pH 4–6), or glycine buffer (glycine/HCl, pH 2–4) at a concentration of 5 mg/mL. The optical transmittance of the solutions at 600 nm was recorded using a V-550 UV/VIS spectrophotometer (Jasco, Tokyo, Japan).

### 2.6. Quartz crystal microbalance with dissipation monitoring (QCM-D)

Lipid bilayer-coated sensor chips were prepared as previously described [38,39]. Briefly, 1,2-dioleoyl-sn-glycero-3-phosphocholine (DOPC; Avanti Polar Lipid, Alabaster, AL, USA) was dissolved in chloroform at a concentration of 10 mg/mL. Subsequently, lipid solution was completely dried with nitrogen gas to form a lipid film. To the lipid film, 2-[4-(2-hydroxyethyl)piperazin-1-yl]ethanesulfonic acid (HEPES) buffer (10 mM HEPES, 150 mM NaCl, pH 7.4; 5 mL) was added to the lipid film and sonicated to disperse the lipids. The solution was then extruded 21 times through 100-nm membranes using a mini extruder (Avanti Polar Lipid) for obtaining a lipid vesicle solution. QCM-D monitoring was performed using a QSence E1 (Biolin Scientific AB, Gothenburg, Sweden). The QSX 303 SiO_2_ sensor (Biolin Scientific AB) was mounted on the QCM-D flow modules. A peristaltic pump (Reglo Digital M2-2/12, Ismatec, Wertheim, Germany) was used for injecting degassed HEPES buffer at a flow rate of 100 μL/min until the signal drift was less than 1 Hz for the frequency shift and 0.2 × 10^−6^ for the dissipation shift in 10 min. Next, the vesicle solution was injected into the chamber at a flow rate of 100 μL/min for 15 min for constructing a lipid layer on the SiO_2_ sensor chip surfaces. The sensor chip surfaces were then rinsed with degassed HEPES buffer for 10 min to remove excess vesicles. For the lipid-coated sensor ships, the carboxylated PRX solutions (1 mM threaded β-CD in HEPES buffer) were injected for 5 min at a flow rate of 100 μL/min, followed by stopping of the flow and incubation for 10 min to the allow carboxylated PRXs to be adsorbed onto the lipid surfaces. Finally, the sensor chip surfaces were rinsed with phosphate buffer saline (PBS, pH 7.4) for 10 min at a flow rate of 100 μL/min for removing free carboxylated PRXs, and the shifts in frequency (ΔF) and dissipation (ΔD) were determined.

Protein-immobilized sensor chips were prepared as described previously [40]. Briefly, QSX 301 gold sensor chips (Biolin Scientific AB) were immersed in a cysteamine (Merck) solution (10 mM in ethanol) overnight at room temperature. The sensor chips were subsequently rinsed with distilled water, dried with nitrogen gas, and mounted on the QCM-D flow modules. A peristaltic pump was used for injecting PBS at a flow rate of 50 μL/min until the signal drift was stable. Next, 2% glutaraldehyde solution in PBS (Merck) was injected into the flow modules at a flow rate of 50 μL/min for 30 min, followed by a rinse with PBS for 20 min for removing unreacted glutaraldehyde. Bovine serum albumin (BSA; Fujifilm Wako Pure Chemical) dissolved in PBS (10 mg/mL) was passed into the chamber for 30 min at a flow rate of 50 μL/min, followed by stopping of the flow and incubation for 60 min to allow immobilization of the BSA onto the sensor chip surfaces. After rinsing of the surfaces with PBS for 20 min, the carboxylated PRX solutions (1 mM threaded β-CD in HEPES buffer) were injected for 20 min at a flow rate of 50 μL/min, followed by stopping of the flow and incubation for 70 min to allow adsorption of the carboxylated PRXs onto the BSA-immobilized surfaces. Finally, the sensor chip surfaces were rinsed with PBS for 30 min at a flow rate of 50 μL/min to remove free carboxylated PRXs, and the ΔF and ΔD were determined.

### 2.7. Cell culture

RAW 264.7 cells, a mouse macrophage-like cell line, was obtained from the American Type Culture Collection (ATCC, Manassas, VA, USA). NIH/3T3 cells, a mouse embryonic fibroblast cell line, was obtained from the Japanese Collection of Research Bioresources (JCRB, Osaka, Japan). RAW264.7 and NIH/3T3 cells were cultured in Dulbecco’s modified Eagle’s medium (DMEM) (Fujifilm Wako Pure Chemical) supplemented with 10% fetal bovine serum (FBS; Gibco, Grand Island, NY, USA), 100 units/mL penicillin (Fujifilm Wako Pure Chemical), and 100 µg/mL streptomycin (Fujifilm Wako Pure Chemical) in 5% CO_2_ at 37°C.

### 2.8. Expression level of macrophage scavenger receptor

Cells were stained with allophycocyanin (APC)-labeled rat anti-mouse MSR-A (CD204) antibody (APC-anti-MSR-A; clone: M204PA; Thermo Fisher Scientific, Waltham, MA, USA) or APC-labeled rat IgG2a,κ isotype control antibody (clone: eBR2a; Thermo Fisher Scientific) for 30 min on ice. The Fc receptors in RAW 264.7 cells were blocked with TruStain FcX (anti-mouse CD16/32; clone:93; BioLegend, San Diego, CA, USA) for 30 min on ice before the cells were labeled with antibodies. The stained cells were subsequently collected through centrifugation at 200 × *g* at 4°C for 3 min, washed twice with PBS containing 0.1% BSA, and passed through a 35-μm cell strainer (Corning, Corning, NY, USA). The fluorescence intensity of the cells was measured using a NovoCyte 2000 flow cytometer (ACEA Biosciences, San Diego, CA, USA). The stained cells were excited using a 640-nm laser and detected using a 675 ± 30-nm bandpass filter. A total of 10,000 cells were counted for each sample, and the fluorescence intensity of the cell population was determined using the Novo Express software (version 1.2.5, ACEA Biosciences).

### 2.9. Immunoblotting

Cells were lysed with radioimmune precipitation assay (RIPA) buffer (Fujifilm Wako pure Chemical) containing Complete Protease Inhibitor Cocktail (Roche, Basel, Switzerland). The lysates were clarified through centrifugation at 15,000 rpm for 10 min, and the supernatant was collected. SDS-PAGE was performed on a 10% polyacrylamide gel for 50 min at 150 V. The samples were subsequently transferred to a poly(vinylidene difluoride) (PVDF) membrane (Bio-Rad, Hercules, CA, USA) using a Trans-Blot Turbo transfer system (Bio-Rad). The membrane was blocked with TBST buffer (20 mM Tris-HCl, 500 mM NaCl, 0.05% Tween 20, pH 7.5) containing 5% BSA for 60 min at room temperature. The membrane was subsequently incubated overnight at 4°C with rat monoclonal MSR-A (clone: 7G5C33; BioLegend; 1:200 dilution) and rabbit polyclonal anti-β-actin (A2066; Merck; 1:1,000 dilution). The membrane was treated with horseradish peroxidase (HRP)-conjugated goat anti-rat IgG (catalog number: 7077; Cell Signaling Technologies, Danvers, MA, USA; 1:1000 dilution) or goat anti-rabbit IgG (catalog number: 7074; Cell Signaling Technologies; 1:1000 dilution) for 60 min at room temperature. The membrane was treated using ImmunoStar Zeta (Fujifilm Wako Pure Chemical). Chemiluminescence images were acquired using an ImageQuantLAS500 imager (GE Healthcare, Chicago, IL, USA).

### 2.10. Cellular uptake analysis by flow cytometry

For the analysis of cellular internalization of carboxylated PRXs, BODIPY-modified carboxylated PRXs and HEE-PRX were synthesized as described in the **Supplementary text**. RAW 264.7 and NIH/3T3 cells were plated in a 24-well plate at a density of 1 × 10^5^ and 5 × 10^4^ cells/well, respectively, and incubated overnight. BODIPY-labeled carboxylated PRXs and HEE-PRX solutions (200 μM threaded β-CD) were applied to the cells and incubated at 37°C for 24 h. The cells were harvested, collected by centrifugation at 200 × g at 4°C for 3 min, washed twice with PBS containing 0.1% BSA, and passed through a 35-μm cell strainer. The fluorescence intensity of the treated cells was measured using a NovoCyte 2000 flow cytometer. The FITC-labeled PRX-treated cells were excited using a 488-nm laser and detected using a 530 ± 30-nm bandpass filter. The fluorescence intensity of the treated cells was analyzed as described above.

To investigate the involvement of MSR-A in the cellular uptake of BODIPY-labeled carboxylated PRXs and HEE-PRX, the cells were pre-treated with dextran sodium sulfate (DSS; molecular weight = 5,000; 0.1–10 mM sulfate group; Fujifilm Wako Pure Chemical) for 3 h at 37°C. Subsequently, the cells were treated with BODIPY-labeled carboxylated PRXs and HEE-PRX for 24 h at 37°C in the presence of DSS. The fluorescence intensity of the treated cells was analyzed by flow cytometry as described above.

### 2.11. Intracellular distribution analysis

The cells were plated in a 35-mm glass bottom dish (diameter of glass area: 12 mm; Iwaki, Tokyo, Japan) at a density of 1 × 10^4^ cells/dish and incubated overnight. Next, they were cultured in a treatment medium containing BODIPY-labeled carboxylated PRXs and HEE-PRX for 24 h at 37°C. Subsequently, the cells were stained with 100 nM LysoTracker Red DND-99 (Thermo Fisher Scientific) at 37°C for 15 min, followed by staining with 1 μg/mL Hoechst 33,342 (Dojindo Laboratories, Kumamoto, Japan) at 37°C for 10 min. Confocal laser scanning microscopy (CLSM) was performed using a FluoView FV10i (Olympus, Tokyo, Japan) equipped with a 60× water-immersion objective lens (numerical aperture: 1.2). The excitation and emission wavelengths for Hoechst 33,342, BODIPY-labeled PRXs, and LysoTracker Red were 405 nm and 455 nm, 473 nm and 520 nm, and 559 nm and 598 nm, respectively. The co-localization of BODIPY-labeled PRXs with LysoTracker-stained puncta was quantified using Fiji’s Coloc 2 plugin for calculating Pearson’s correlation coefficient [41].

### 2.12. Cell viability

The cells were plated on a 96-well plate at a density of 1 × 10^4^ cells/well and incubated overnight. Subsequently, the carboxylated PRXs (1–1,000 μM threaded β-CD) were added to each well. After incubation for 24 h, Cell Counting Kit-8 reagent (Dojindo Laboratories) was added to each well according to the manufacturer’s instructions. After incubation for 1.5 h at 37°C, the absorbance was measured on a Varioskan LUX multimode microplate reader (Thermo Scientific) at 450 nm. Cellular viability was calculated by comparing the absorbance of the treated cells to that of the untreated cells.

### 2.13. Live/dead staining

RAW 264.7 and NIH/3T3 cells were plated in a 24-well plate at a density of 1 × 10^5^ and 5 × 10^4^ cells/well, respectively, and incubated overnight. Subsequently, the cells were cultured in a treatment medium containing carboxylated PRXs (10 mM threaded β-CD) for 24 h at 37°C. Next, they were stained using a Live/Dead Cell Staining Kit II (PromoCell, Heidelberg, Germany) for 30 min at 37°C; in this procedure, calcein-AM (2 μM) and ethidium homodimer III (EthD III; 4 μM) stained live and dead cells, respectively. The phase contrast and fluorescent images of the stained cells were acquired using an IX-71 (Olympus) equipped with a DP-80 dual CCD microscope camera.

### 2.14. Statistical analysis

Statistical analyses were performed using OriginPro 2021 (OriginLab, Northampton, MA, USA). The statistical differences between the means of individual groups were determined using one-way analysis of variance (ANOVA) followed by the Tukey-Kramer multiple comparison test. A *P* value less than 0.05 was considered statistically significant.

## 3. Results and discussion

### 3.1. Synthesis and characterization of carboxylated PRXs

Previously reported methods for the carboxylation of non-degradable α-CD PRXs are reactions with succinic anhydrides [42–44], glycine [45], and bromoacetic acid [46,47]. However, these methods cannot avoid acidification of the solutions, and they are difficult to be applied for the carboxylation of acid-degradable β-CD PRXs. We reported the synthesis of carboxyethyl ether-modified non-degradable α-CD PRXs using methyl 3-bromopropionate under strongly alkaline conditions [34]. Because this reaction proceeds under alkaline conditions, we first tried to synthesize acid-degradable carboxylated β-CD PRXs using methyl 3-bromopropionate. However, we failed to modify the carboxyethyl ether on acid-degradable β-CD PRXs. It is likely that the methyl ester in methyl 3-bromopropionate is hydrolyzed in alkaline conditions and the resulting 3-bromopropionic acid may cause the degradation of the β-CD PRXs. Therefore, we attempted to modify carboxy groups onto acid-degradable β-CD PRXs through two-step reactions, as shown in Figure 2. The first step is the modification of glycine ethyl ester using CDI, which is generally utilized for the chemical modification of PRXs [7,13,16,17,45]. The second step is the deprotection of the esters under mildly alkaline conditions. This two-step method avoids the acidification of the solutions and the decomposition of acid-degradable PRXs. Additionally, this method allows the preparation of various carboxylated β-CD PRXs with different alkyl spacer lengths between carboxy groups and threaded β-CDs (Figures 1 and 2). Note that the carbamate linkages between β-CD and carboxyalkyl groups do not interfere with the inclusion ability of cholesterol and other compounds [7,48], and carboxylated β-CDs are utilized for cholesterol binding [49,50]. Therefore, we considered that the carboxyalkyl carbamates-modified β-CD PRXs are applicable to the treatment of cholesterol-related metabolic diseases via the inclusion of intracellular cholesterol. In this study, three series of carboxylated β-CD PRXs with different alkyl spacer lengths were synthesized for verifying the effects of the alkyl spacer length adjacent to the carboxy groups on the cellular internalization efficiency of the carboxylated β-CD PRXs.

**Figure 3.**
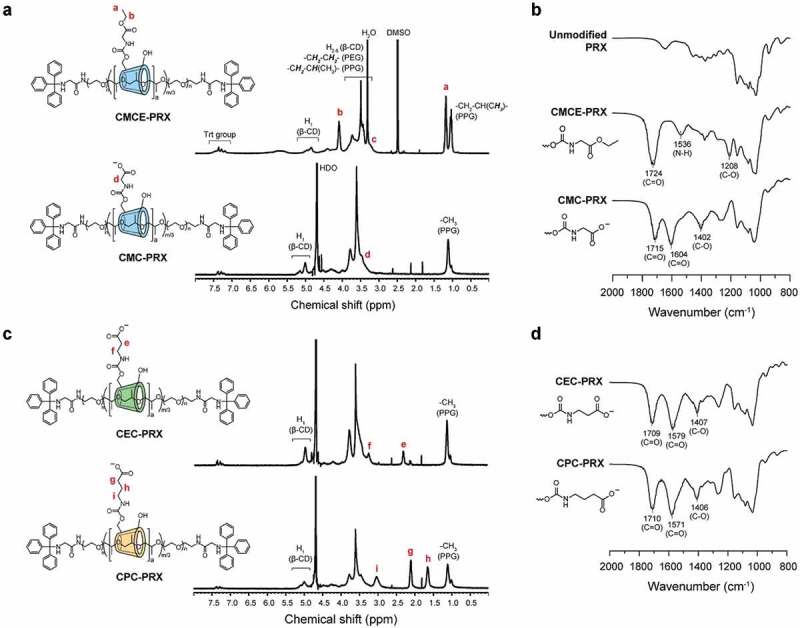
(a) ^1^H NMR spectra of CMCE-PRX in DMSO-*d*_6_ and CMC-PRX in D_2_O (400 MHz, 25°C). (b) FT-IR spectra of unmodified PRX, CMCE-PRX, and CMC-PRX. (c) ^1^H NMR spectra of CEC-PRX and CPC-PRX in D_2_O (400 MHz, 25°C). (d) FT-IR spectra of CEC-PRX and CPC-PRX

The modification of CMCE on the PRXs was characterized through ^1^H NMR and FT-IR measurements. For quantifying the number of modified CMCEs on PRXs by ^1^H NMR, CMCE-PRX was synthesized using glycine ethyl ester (in the case of glycine methyl ester, the peak of methyl ester overlapped with the peaks of β-CD). In the ^1^H NMR spectrum of CMCE-PRX (Figure 3(a)), new peaks assignable to ethyl ester moieties appeared at 1.18 ppm (-O-CH_2_-C***H_3_***) and 4.08 ppm (-O-C***H_2_***-CH_3_). Additionally, in the FT-IR spectrum of CMCE-PRX (Figure 3(b)), new peaks assignable to carbamate moieties appeared at 1208 cm^−1^ (C–O–C stretching vibration), 1536 cm^−1^ (N-H bending vibration), and 1724 cm^−1^ (C = O stretching vibration). These results confirm that the CMCE groups were successfully modified onto PRXs. Similarly, in the ^1^H NMR spectra of carboxyethyl carbamate methyl ester-modified PRX (CECM-PRX) and carboxypropyl carbamate methyl ester-modified PRX (CPCM-PRX), new peaks assignable to alkyl spacers were observed at 2.45 ppm for CECM-PRX and at 1.64 and 2.45 ppm for CPCM-PRX (Supplementary Figure S2). The FT-IR spectra of CECM-PRX and CPCM-PRX showed new peaks assignable to carbamate moieties (Supplementary Figure S3). Therefore, modification of the carboxy esters was not affected by the alkyl spacer length. Additionally, after modification with carboxy esters, no peaks corresponding to free β-CD and axle polymer were observed in the SEC charts of CMCE-PRX, CECM-PRX, and CPCM-PRX (Supplementary Figure S4), indicating that carboxy esters were successfully modified in the PRXs without degradation.

The deprotection of ester moieties was completed within 6 h in 0.5 M NaOH solutions. The ^1^H NMR spectrum of the deprotected product (CMC-PRX) confirmed that the peaks assignable to ethyl ester were completely disappeared (Figure 3(a)). The same results were confirmed for CEC-PRX and CPC-PRX (Figure 3(c)). In the FT-IR spectrum of CMC-PRX, new peaks were observed at 1604 and 1402 cm^−1^, which correspond to the carboxylate anion [47]. The same tend was observed in the FT-IR spectra of CEC-PRX and CPC-PRX (Figure 3(d)). We considered that the carboxylated PRXs were obtained as an ammonium salt, because these PRXs were purified by dialysis in ammonium solution for maintaining the pH at weakly alkaline conditions. The number of modified carboxy groups on the PRXs and number-average molecular weights are summarized in Table 1. The number of modified carboxy groups in CMC-PRX, CEC-PRX, and CPC-PRX were roughly identical (approximately 2 carboxy groups were modified per threaded β-CD). In addition to carboxylated PRXs, we synthesized HEE-PRX as a control for the cell experiments (Table 1). The number of modified HEE groups was 4.0 per threaded β-CD, because at least 4 HEE groups are required for dissolution in aqueous solutions [7].

**Table 1.** Characterization of carboxylated PRXs used in the present study

Code	Number of threaded β-CDs	Number of functional groups on PRX (per β-CD) ^a^		*M*_n_^b^
		Carboxy	HEE	
CMC-PRX	12.5	31.3 (2.5)	-	24,200
CEC-PRX	12.5	27.5 (2.2)	-	24,100
CPC-PRX	12.5	22.5 (1.8)	-	23,800
HEE-PRX	12.5	-	50.0 (4.0)	27,100

^a^Determined using ^1^H NMR in DMSO-*d*_6_ or D_2_O at 25°C. The values in parentheses denote the average number of modified functional groups per β-CD threaded on PRXs. ^b^Calculated on the basis of the chemical composition of the modified PRXs, as determined using ^1^H NMR. The *M*_n_ of the modified PRXs was calculated as an ammonium salt.

### 3.2. *p*K_a_ *and pH-responsivity of carboxylated β-CD PRXs*

**Figure 4.**
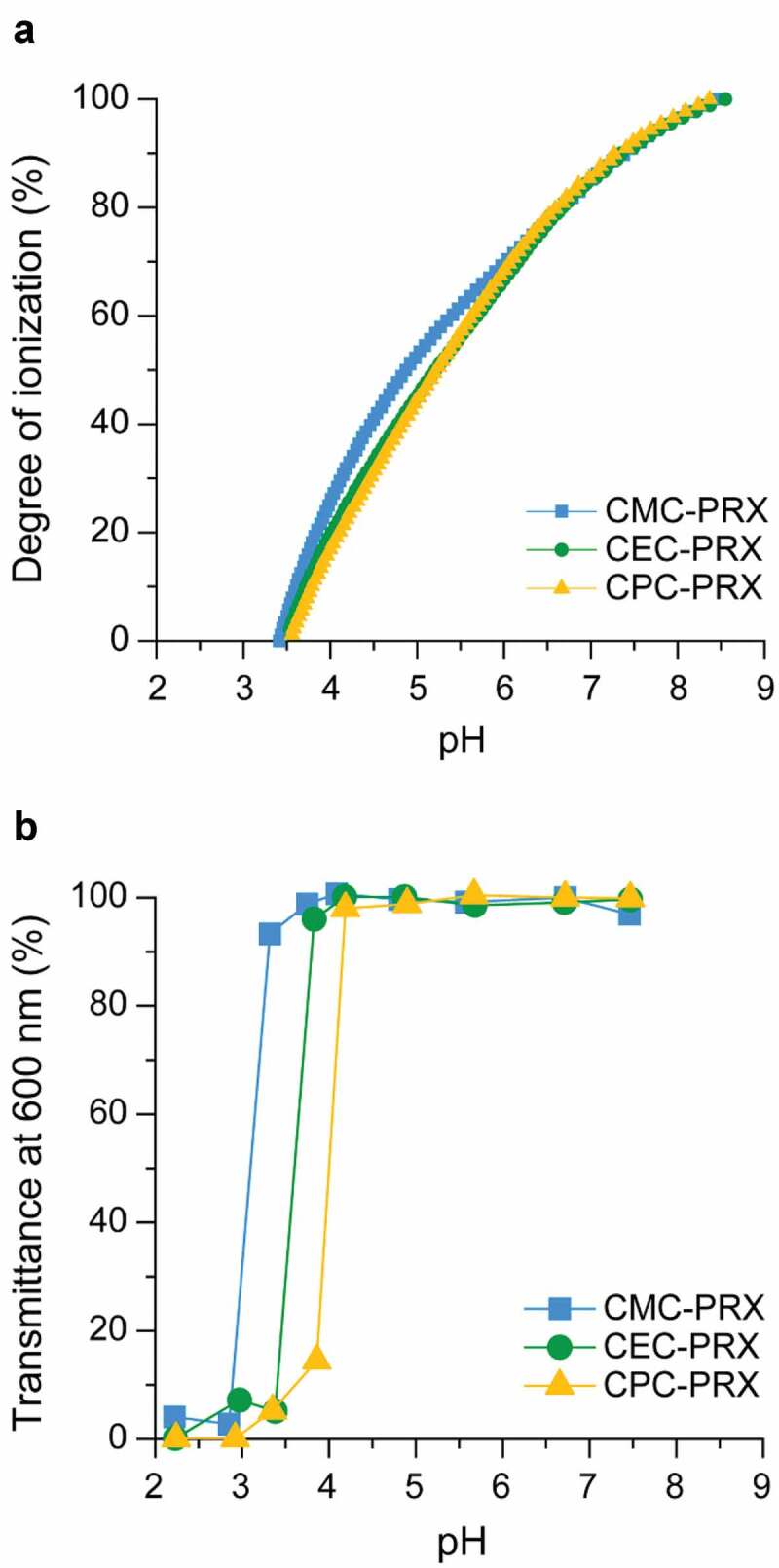
(a) Relationship between the pH and degree of ionization of carboxy groups in CMC-PRX (squares), CEC-PRX (circles), and CPC-PRX (triangles), as determined using potentiometric titration in 10 mM NaCl at 25°C. (b) Relationship between the pH and transmittance of CMC-PRX (squares), CEC-PRX (circles), and CPC-PRX (triangles) solutions (5 mg/mL) at 25°C

The ionization degree of each carboxylated β-CD PRX was evaluated by potentiometric titration. The acid dissociation constant (p*K*_a_) of the carboxy groups in each carboxylated β-CD PRXs were determined at an ionization degree of 50% on the basis of the pH–ionization degree curves, as shown in Figure 4(a), and Table 2. Upon increasing alkyl spacer length, the p*K*_a_ values of the carboxy groups increased slightly. Because electron-donating alkyl substituents decrease the acidity of carboxylic acids, the trend of the p*K*_a_ values for the carboxylated β-CD PRXs is reasonable. From the pH–ionization degree curves, the ionization degree of the carboxy groups at pH 7.4 was also determined (Table 2). Under physiological pH conditions, approximately 90% of the carboxylic acids were ionized for all the carboxylated β-CD PRXs.

**Table 2.** p*K*_a_ of carboxylated PRXs

Code	p*K*_a_^a^	Degree of ionization at pH 7.4 (%)
CMC-PRX	4.87	90.0
CEC-PRX	5.21	90.4
CPC-PRX	5.27	90.9

^a^Determined using potentiometric titration in 10 mM NaCl at 25°C.

Next, the solubility of carboxylated β-CD PRXs at various pH conditions was tested by dissolving each carboxylated β-CD PRX in a buffer solution. Each carboxylated β-CD PRX solution was transparent at a pH range of 7.4 to 4, whereas precipitation of the carboxylated β-CD PRXs was observed below pH 4 (Supplementary Figure S5), which was most likely due to the protonation of carboxy groups under acidic pH conditions. Subsequently, the optical transmittance of each carboxylated β-CD PRX solution was recorded (Figure 4(b)). Although the transmittance of the solutions decreased under acidic pH conditions, upon increasing the alkyl spacer length in carboxylated β-CD PRXs, the threshold pH values at which the transmittance decreased were shifted to higher pH values. These threshold pH values were not consistent with the p*K*_a_ values of each carboxylated β-CD PRX. Because the hydrophobicity of the modified carboxy groups increases with the alkyl spacer length, CPC-PRX precipitated at a high pH. Accordingly, these results indicate that the carboxylated β-CD PRXs do not precipitate even in the lysosomes, because the pH value in the lysosomes is approximately 4.5 to 5.0 [51].

### 3.3. Interaction of carboxylated β-CD PRX with lipid and protein

**Figure 5.**
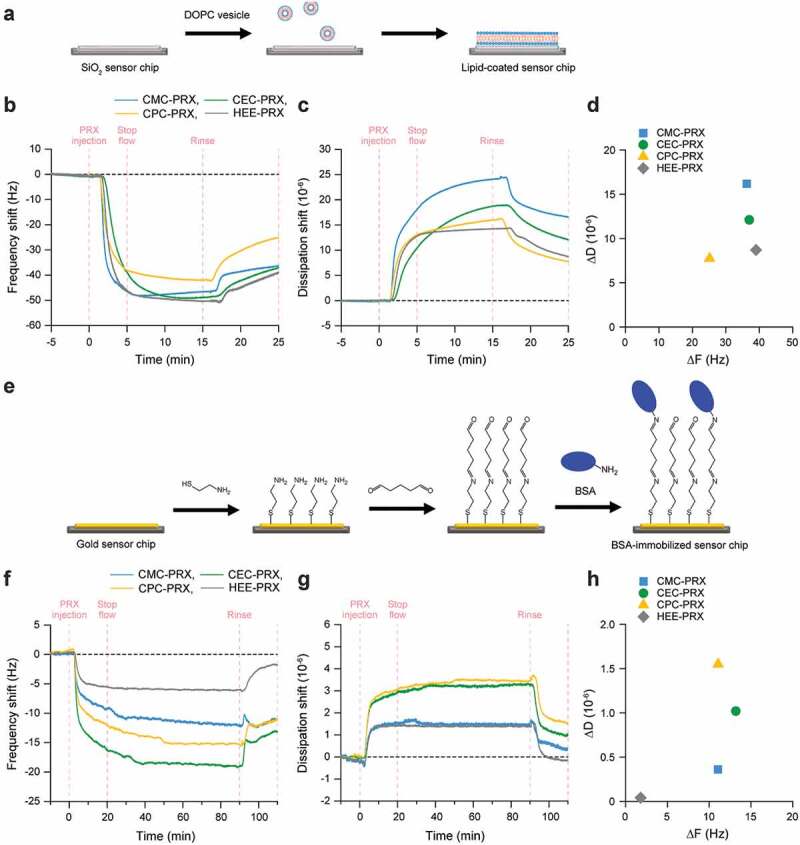
(a) Schematic illustration for the construction of DOPC lipid layer on SiO_2_ sensor chip surfaces. (b-d) QCM-D monitoring of the interaction of carboxylated PRXs and HEE-PRX with DOPC lipid layer: frequency shift (b), dissipation shift (c), and ΔF vs ΔD plot (d). (e) Schematic illustration for the immobilization of BSA on gold sensor chip surfaces. (F-H) QCM-D monitoring of the interaction of carboxylated PRXs and HEE-PRX with BSA-immobilized surfaces: frequency shift (f), dissipation shift (g), and ΔF vs ΔD plot (H)

To assess the interaction of carboxylated PRXs with lipid and proteins, QCM-D measurements were performed using lipid-coated and protein-immobilized sensor chips. The DOPC lipid layer was constructed on the sensor chip surfaces (Figure 5(a)) [38,39]. The frequency shifts on DOPC lipid layers flowed with carboxylated PRX and HEE-PRX solutions were almost comparable (Figure 5(b)); however, CPC-PRX showed fewer frequency shifts. The dissipation shifts of CMC-PRX and CEC-PRX were slightly larger than those of HEE-PRX (Figure 5(c)). Accordingly, the interaction of carboxylated PRXs with the lipid layer was slightly lower than that of HEE-PRX. Because the zeta potential of the DOPC lipid layer is slightly negative (−10 to 0 mV) [52], it is considered that the carboxylated PRXs showed relatively weak interactions with the DOPC lipid layer owing to electrostatic repulsion. When ΔF and ΔD were plotted (Figure 5(d)), ΔD/ΔF of carboxylated PRXs was larger than that of HEE-PRX, suggesting that the carboxylated PRXs were loosely adsorbed onto the lipid layer.

The interaction of carboxylated PRXs with protein was assessed using BSA as a protein model, and BSA was immobilized on the sensor chip surfaces (Figure 5(e)). The frequency shifts on BSA-immobilized surfaces flowing with carboxylated PRX solutions were obviously greater than those of HEE-PRX (Figure 5(f)), and the dissipation shifts of carboxylated PRXs were large compared with that of HEE-PRX (Figure 5(g)). Although BSA is a negatively charged protein (pI = 4.9) [15], all the carboxylated PRXs showed significantly high ΔF with BSA, probably due to the electrostatic interactions with cationic amino acid moieties in BSA. However, when ΔF and ΔD were plotted, CEC-PRX and CPC-PRX showed high values of ΔD/ΔF compared with CMC-PRX and HEE-PRX (Figure 5(h)), suggesting that CEC-PRX and CPC-PRX loosely interact with BSA. Consequently, it is plausible that the carboxylation of β-CD PRXs increases their interaction with proteins, whereas it slightly decreases their interaction with lipids.

### 3.4. Intracellular uptake of carboxylated β-CD PRX

**Figure 6.**
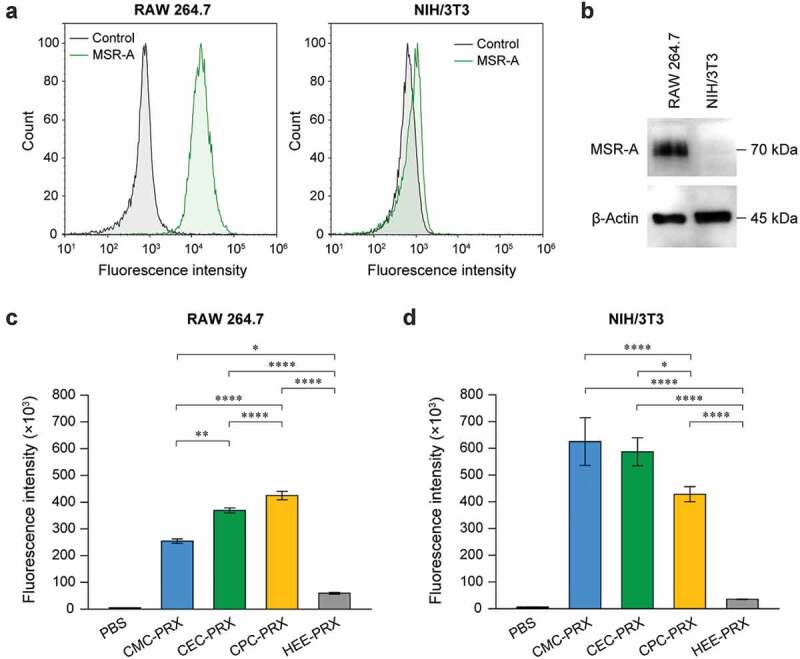
(a) Fluorescence intensity histograms of RAW 264.7 and NIH/3T3 cells treated with APC-anti-MSR-A antibody or isotype control. (b) Immunoblot analysis for MSR-A and β-actin in RAW 264.7 and NIH/3T3 cells. (c, d) Fluorescence intensity of RAW 264.7 (c) and NIH/3T3 cells (d) treated with BODIPY-labeled carboxylated PRX (200 μM threaded β-CD; CMC-PRX = 387 μg/mL, CEC-PRX = 386 μg/mL, CPC-PRX = 381 μg/mL) and HEE-PRX (200 μM threaded β-CD; 432 μg/mL) for 24 h. The data are expressed as the mean ± standard deviation (SD) (n = 3; *P < 0.05, **P < 0.01, ***P < 0.001, ****P < 0.0001)

We have previously reported that carboxylated non-degradable α-CD PRXs showed higher intracellular uptake into RAW 264.7 cells compared with HEE-modified α-CD PRX through molecular recognition by MSR-A [34], because the cysteine-rich domain at the extracellular C-terminus of MSR-A recognizes anionic macromolecules [35]. To investigate the cellular uptake level of each carboxylated β-CD PRX and the involvement of MSR-A in their cellular uptake, RAW 264.7 and NIH/3T3 cells were used as MSR-A-positive and MSR-A-negative cells, respectively. Flow cytometry and immunoblot analysis confirmed the expression of MSR-A in RAW 264.7 cells, whereas MSR-A expression was negligible in NIH/3T3 cells (Figure 6(a,b)).

The intracellular uptake levels of carboxylated β-CD PRXs in RAW 264.7 and NIH/3T3 cells were evaluated using flow cytometry and BODIPY-labeled carboxylated β-CD PRXs (Supplementary Table S1). BODIPY-labeled HEE-PRX was utilized as a control. Note that the modification of BODIPY to PRXs may alter the physicochemical properties and intracellular uptake, the number of modified BODIPY on PRXs was minimized to avoid the effect of BODIPY (0.1 to 0.2 BODIPY molecules per PRX). In RAW 264.7 cells, the intracellular uptake of all carboxylated β-CD PRXs were significantly higher than that of HEE-PRX (Figure 6(c), Supplementary Figure S6), which is consistent with the previous result on carboxylated α-CD PRX [34]. Additionally, the intracellular uptake of the carboxylated β-CD PRXs was ranked in the following order: CPC-PRX > CEC-PRX > CMC-PRX. The intracellular uptake level of carboxylated α-CD PRX in RAW 264.7 cells was changed by structural parameters such as the number of modified carboxy groups and the molecular weight of the axle polymer, most likely due to the difference in recognition by MSR-A [34]. Therefore, the results suggest that the length of alkyl spacers adjacent to carboxy groups affects the intracellular uptake efficiency and that CPC groups are suitable for the uptake of acid-degradable β-CD PRXs into RAW 264.7 cells.

In NIH/3T3 cells, the cellular internalization levels of carboxylated β-CD PRXs were expected to be comparable to that of HEE-PRX because NIH/3T3 cells do not express MSR-A. In fact, the carboxylated α-CD PRXs showed comparable intracellular uptake to HEE-modified α-CD PRX in NIH/3T3 cells [34]. However, the intracellular uptake of all the carboxylated β-CD PRXs in NIH/3T3 cells was significantly higher than that of HEE-PRX (Figure 6(d), Supplementary Figure S6). The intracellular uptake levels of carboxylated β-CD PRXs are ranked in the following order: CMC-PRX > CEC-PRX > CPC-PRX, which was opposite to the results for RAW 264.7 cells. Additionally, the fluorescence intensities of NIH/3T3 cells treated with carboxylated β-CD PRXs were greater than those of RAW 264.7 cells. This is most likely due to the difference in the size and the extent of endocytosis between RAW 264.7 and NIH/3T3 cells.

We additionally evaluated the intracellular uptake of carboxylated β-CD PRXs in DC2.4 cells, a mouse dendritic cell line, which also did not express MSR-A (Supplementary Figure S7). The intracellular uptake of all the carboxylated β-CD PRXs were significantly higher than that of HEE-PRX in the DC2.4 cells; however, the order of intracellular uptake was different from that in NIH/3T3 cells (Supplementary Figure S7). These results indicate that the modification of carboxyalkyl carbamates in acid-degradable β-CD PRXs facilitates intracellular uptake, even in MSR-A-negative cells.

**Figure 7.**
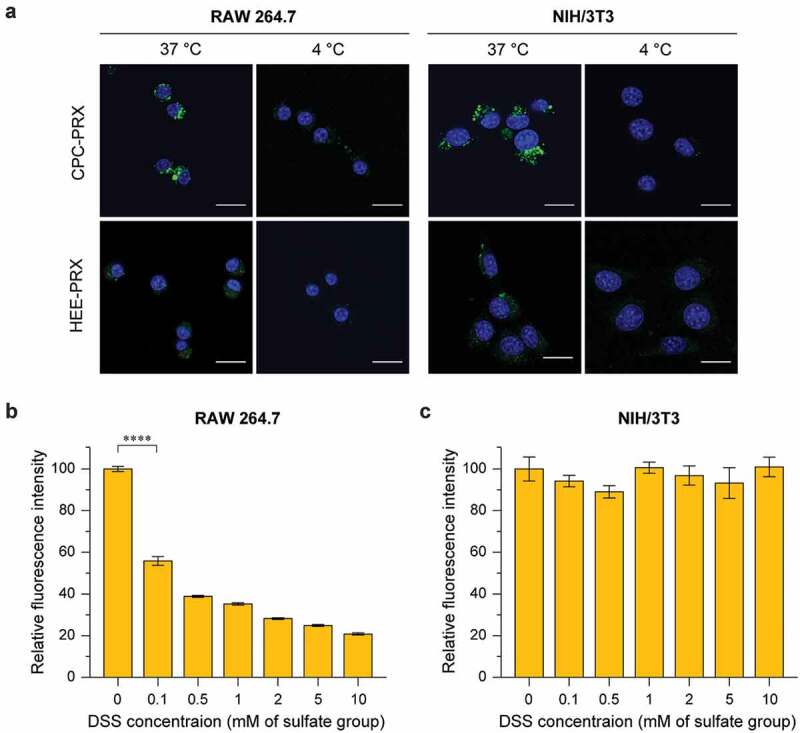
(a) CLSM images of RAW 264.7 and NIH/3T3 cells treated with BODIPY-labeled CPC-PRX (green; 200 μM threaded β-CD) and HEE-PRX (green; 200 μM threaded β-CD) for 1 h at 37°C (Scale bars: 20 μm). The cell nuclei (blue) were stained with Hoechst 33,342. (b, c) Fluorescence intensity of RAW 264.7 (b) and NIH/3T3 cells (c) treated with DSS (0.1 to 10 mM with respect to the sulfate group in DSS) for 3 h at 37°C, followed by treatment with BODIPY-labeled CPC-PRX (200 μM threaded β-CD) and HEE-PRX (200 μM threaded β-CD) for 3 h. The data are expressed as the mean ± SD (n = 3; ****P < 0.0001)

Next, to verify the intracellular uptake pathway of carboxylated β-CD PRXs, we analyzed the intracellular uptake after incubation at 4°C or in the presence of dextran sodium sulfate (DSS), a competitive inhibitor of MSR-A [35]. These experiments were performed for CPC-PRX, because CPC-PRX showed significantly higher uptake in the evaluated cells compared with HEE-PRX. CLSM observation confirmed that the intracellular uptake of BODIPY-CPC-PRX and BODIPY-HEE-PRX was suppressed in RAW 264.7 and NIH/3T3 cells when treated at 4°C for 1 h (Figure 7(a)), suggesting that the intracellular uptake of CPC-PRX follows an energy-dependent endocytosis pathway.

When the cells were pre-treated with DSS for 3 h, the intracellular uptake of BODIPY-CPC-PRX in RAW 264.7 cells was significantly decreased with increasing DSS concentration (Figure 7(b)). This result confirmed that MSR-A was involved in the intracellular uptake of CPC-PRX in RAW 264.7 cells. In contrast, the intracellular uptake of CPC-PRX in MSR-A-negative NIH/3T3 cells was not suppressed by DSS treatment (Figure 7(c)). Because the interaction of carboxylated β-CD PRXs with BSA was stronger than that of HEE-PRX (Figure 5(d)), carboxylated β-CD PRXs might strongly interact with membrane proteins, leading to high intracellular uptake. Although we could not identify the membrane proteins involved in the cellular internalization of carboxylated β-CD PRXs in MSR-A-negative cells, we considered that the carboxyalkyl carbamate moieties in carboxylated β-CD PRXs are recognized by membrane-bound proteins. The carboxyalkyl groups modified on β-CD PRXs are naturally occurring amino acids (CMC = glycine, CEC = β-alanine, CPC = γ-aminobutyric acid: GABA). Because these amino acids and their carbamate derivatives are the substrate of amino acid transporters or membrane-bound acylases [53–55], the interaction of the carboxyalkyl carbamates modified on PRXs to these membrane proteins might contribute to the intracellular uptake in MSR-A-negative cell lines. Consequently, the modification of carboxyalkyl carbamates on β-CD PRXs is effective for promoting not only MSR-A-dependent uptake, but also MSR-A-independent uptake in NIH/3T3 cells and other MSR-A-negative cells. Although it is difficult for carboxylated β-CD PRXs to selectively target macrophages through MSR-A recognition, the high intracellular uptake capability of carboxyalkyl carbamate-modified β-CD PRXs in MSR-A-negative cells is beneficial for their therapeutic applications.

### 3.5. Intracellular distribution of carboxylated β-CD PRX

**Figure 8.**
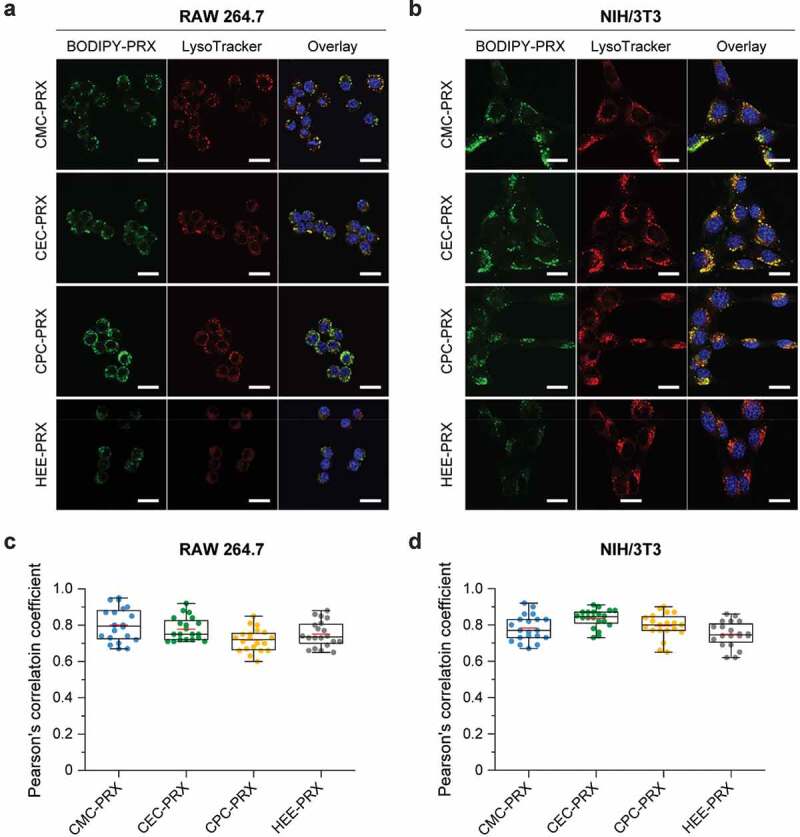
(a, b) CLSM images of RAW 264.7 (a) and NIH/3T3 cells (b) treated with BODIPY-labeled carboxylated PRX (green; 200 μM threaded β-CD) and HEE-PRX (green; 200 μM threaded β-CD) for 24 h at 37°C (Scale bars: 20 μm). The cell nuclei (blue) and endosomes/lysosomes (red) were stained with Hoechst 33,342 and LysoTracker Red, respectively. (c, d) Pearson’s correlation coefficient between BODIPY-labeled PRXs and LysoTracker Red in treated RAW 264.7 (c) and NIH/3T3 cells (d). The data are expressed as a box plot, wherein the upper and lower boundaries of the box indicate the 75th and 25th percentiles, respectively; the black and red lines within the box indicate the median and the mean, respectively, while the whiskers indicate the minimum and maximum (n = 20 cells)

In addition to intracellular uptake, the intracellular distribution of carboxylated β-CD PRXs is an important characteristic, because β-CD PRXs are designed to release threaded β-CDs in lysosomes, especially in the treatment of NPC disease [7,17]. Thus, the intracellular distribution of BODIPY-labeled carboxylated β-CD PRXs was investigated using CLSM in RAW 264.7 and NIH/3T3 cells after 24 h of treatment. To visualize the localization of BODIPY-labeled carboxylated β-CD PRXs, acidic endosomes and lysosomes were stained with LysoTracker Red. The green fluorescence signals derived from BODIPY-labeled PRXs appeared to co-localize with the acidic endosomes and lysosomes stained as red in RAW 264.7 and NIH/3T3 cells (Figure 8(a,b)). For quantitative analysis, Pearson’s correlation coefficient was determined from the CLSM images (Figure 8(c,d)). There was no significant difference among the different types of BODIPY-labeled PRXs in RAW 264.7 and NIH/3T3 cells. Additionally, Pearson’s correlation coefficient values were relatively high, suggesting that all types of BODIPY-labeled PRXs that were internalized into RAW 264.7 and NIH/3T3 cells were primarily localized in the endosomes/lysosomes. The carboxylation of the β-CD PRXs did not affect the intracellular localization in comparison to HEE-PRX. These results suggest that carboxylated β-CD PRXs are capable of releasing threaded β-CDs in the endosomes/lysosomes, which is important in the treatment of NPC diseases [7,17].

### 3.6. Toxicity of carboxylated β-CD PRX

**Figure 9.**
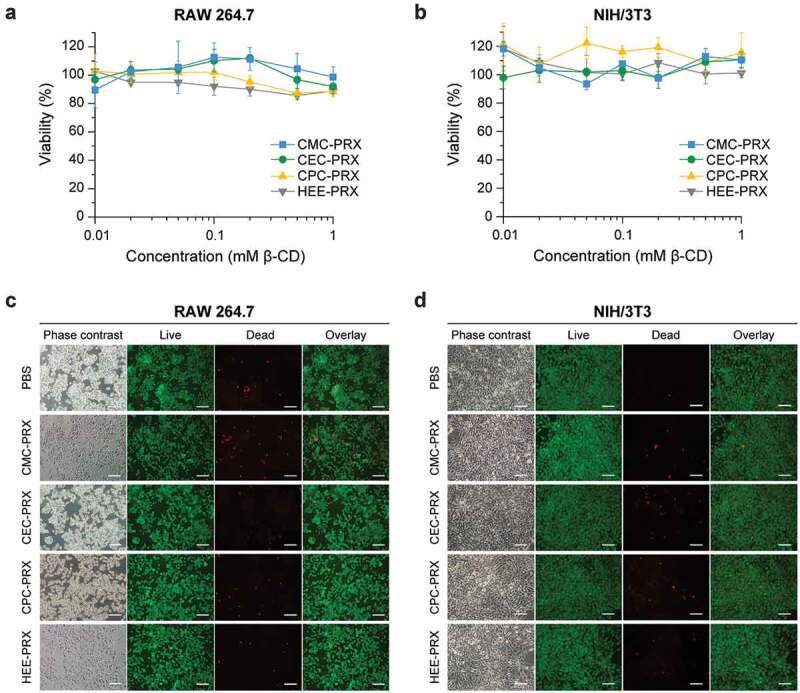
(a, b) Viability of RAW 264.7 (a) and NIH/3T3 cells (b) treated with carboxylated PRXs and HEE-PRX for 24 h. The data are expressed as the mean ± SD (n = 3). (c, d) Phase contrast and fluorescence microscopic images of Live/Dead-stained RAW 264.7 (c) and NIH/3T3 cells (d) treated with carboxylated PRXs (10 mM threaded β-CD; CMC-PRX = 19.4 mg/mL, CEC-PRX = 19.3 mg/mL, CPC-PRX = 19.0 mg/mL) and HEE-PRX (10 mM threaded β-CD; 21.6 mg/mL) for 24 h (scale bars = 100 μm). The live (green) and dead cells (red) were stained with calcein-AM and EthD-III, respectively

The cytotoxicity of a material is an important consideration when designing biomedical materials. In our previous study, HEE-PRX was confirmed to exhibit no cytotoxicity in human fibroblasts [7], because the hydrophobic cavity of β-CDs contains a polymer chain that suppresses the extraction of cholesterol from the plasma membrane [16]. However, the chemical modification of acid-degradable β-CD PRXs with cationic amino groups and methyl groups causes cytotoxicity due to the destabilization of the plasma membrane or the induction of endoplasmic reticulum (ER) stress [16,18]. To confirm whether the carboxylation of β-CD PRXs causes cytotoxicity, we compared the effects of carboxylated β-CD PRX and HEE-PRX on the cell viability of RAW 264.7 and NIH/3T3 cells after 24 h of treatment (Figure 9(a)). When the concentration of threaded β-CDs in carboxylated β-CD PRXs was up to 1 mM, the PRXs did not exhibit cytotoxicity in either the RAW 264.7 or NIH/3T3 cells. These results suggest that the carboxylation of β-CD PRXs does not cause cytotoxicity, regardless of the alkyl spacer length.

To further confirm these results, we tested the cytotoxicity of the carboxylated β-CD PRXs through Live/Dead staining (Figure 9(b)). Most RAW 264.7 and NIH/3T3 cells treated with carboxylated β-CD PRXs (24 h, 10 mM threaded β-CDs) were stained green (live cells); the proportion of dead cells, which were stained red, were comparable to that in the control cells (PBS groups). Consequently, we concluded that the carboxylated β-CD PRXs showed negligible toxicity and that they were suitable candidates for biomedical applications.

## 4. Conclusions

In this study, we developed three series of carboxyalkyl carbamate group-modified β-CD-threaded acid-degradable PRXs with different alkyl spacer lengths to modulate the intracellular uptake efficiency of β-CD PRXs. The carboxylation of β-CD PRXs enabled strong interaction with the protein model, whereas the interactions of these PRXs with the lipid layer model were slightly lower compared with that of HEE-PRX. The carboxylated β-CD PRXs showed significantly high intracellular uptake in MSR-A-positive RAW 264.7 cells compared with HEE-PRX, because the interaction with MSR-A promoted endocytosis of the carboxylated β-CD PRXs. Interestingly, the carboxylated β-CD PRXs showed significantly higher intracellular uptake in the MSR-A-negative NIH/3T3 cells and other cells compared with HEE-PRX. It is plausible that the caboxylated β-CD PRXs strongly interact with membrane proteins, leading to high intracellular uptake regardless of MSR-A expression. Therefore, the carboxylation of β-CD PRXs is an effective chemical modification for promoting both MSR-A-dependent uptake and MSR-A-independent uptake in MSR-A-negative cells. Although it is difficult for carboxylated β-CD PRXs to achieve selective targeting in macrophages, the high intracellular uptake of carboxylated β-CD PRXs in MSR-A-negative cells is beneficial for their therapeutic application. The basic knowledge obtained in this study is expected to serve as an important guide for considering the molecular design of not only PRX, but also CD-based polymers in therapeutic applications [56].

## Supplementary Material

Supplemental Material
