# Supplementary material for: Weakly acidic carboxy group-grafted β-cyclodextrin-threaded acid-degradable polyrotaxanes for modulating protein interaction and cellular internalization
Source: Sci Technol Adv Mater. 2021 Jun 29;22(1):494–510. doi: 10.1080/14686996.2021.1935315 (PMC8245098; doi:10.1080/14686996.2021.1935315)
Supplement: Supplemental Material [file TSTA_A_1935315_SM5755.pdf]

Supplementary data

Weakly acidic carboxy group-grafted β-cyclodextrin-threaded acid-degradable polyrotaxanes for modulating protein interaction and cellular internalization

Shunyao Zhang, Atsushi Tamura*, and Nobuhiko Yui

Department of Organic Biomaterials, Institute of Biomaterials and Bioengineering,
Tokyo Medical and Dental University (TMDU), 2-3-10 Kanda-Surugadai, Chiyoda,
Tokyo 101-0062, Japan.
*Corresponding author: Dr. Atsushi Tamura (E-mail: tamura.org@tmd.ac.jp)

Table of contents

S1. Synthesis of primary amine-terminated Pluronic P123
S2. Synthesis of acid-degradable β-CD PRX
S3. Cell culture
S4. Synthesis of fluorescently labeled carboxylated polyrotaxanes
S5. References

S1. Synthesis of primary amine-terminated Pluronic P123

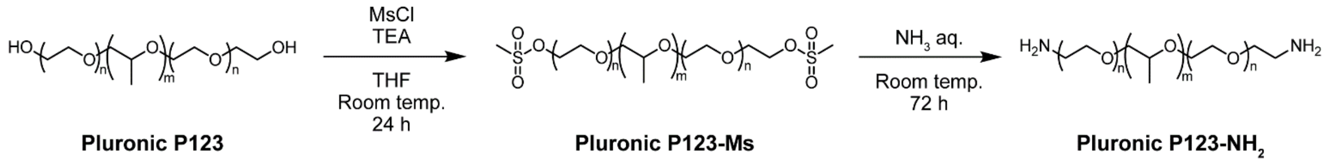

Scheme S1. Synthesis scheme for mesyl- and primary amine-terminated Pluronic P123, where *n* and *m* denote the number of monomer units in PEG and PPG segments, respectively.

Pluronic P123 (the number of monomer units in PEG and PPG segments was n = 19.5 × 2 and m = 66.3, respectively; $M_{n,\text{PEG-}b\text{-PPG-}b\text{-PEG}}$ = 5,570; 20.0 g, 3.59 mmol; Merck, Darmstadt, Germany) was dissolved in dehydrated tetrahydrofuran (THF; 100 mL; Fujifilm Wako Pure Chemical, Osaka, Japan). Triethylamine (TEA; 3.94 mL, 53.87 mmol; Fujifilm Wako Pure Chemical) and methane sulfonyl chloride (MsCl; 4.17 mL, 53.87 mmol; Fujifilm Wako Pure Chemical) were successively added to the solution under a nitrogen atmosphere. The reaction mixture was stirred for 24 h at room temperature, and subsequently filtered and concentrated using a rotary evaporator. The raw product was purified by dialysis against methanol for 2 days using a Spectra/Por 6 dialysis membrane (molecular weight cut-off of 1,000; Spectrum Laboratories, CA, USA). Finally, methanol was removed using a rotary evaporator to yield mesyl-terminated Pluronic P123 (Pluronic P123-Ms; 14.16 g, 68.9% yield). ^{1}H NMR (400 MHz, $CDCl_3$; Figure S1): δ = 1.05 (m, -C$\boldsymbol{H_3}$ of Pluronic P123), 3.08 (s, 3H, -O-SO_2-C$\boldsymbol{H_3}$), 3.1-3.9 (m, -C$\boldsymbol{H_2}$-C$\boldsymbol{H_2}$-O- and -C$\boldsymbol{H_2}$-C$\boldsymbol{H}$(CH_3)- of PEG and PPG in Pluronic P123), 3.76 (t, 2H, -C$\boldsymbol{H_2}$-CH_2-O-SO_2-), 4.38 (t, 2H, -CH_2-C$\boldsymbol{H_2}$-O-SO_2-).

Pluronic P123-Ms (10 g, 1.75 mmol) was dissolved in 28% ammonia solution (50 mL; Kanto Chemical, Tokyo, Japan), and the solution was stirred for 72 h at room temperature. The product was purified by dialysis against water for 3 days using a Spectra/Por 6 dialysis membrane. Finally, the purified product was freeze-dried to yield primary amine-terminated Pluronic P123 (Pluronic P123-NH_2; 6.08 g, 62.5% yield). ^{1}H NMR (400 MHz, $CDCl_3$; Figure S1): δ = 1.05 (m, -C$\boldsymbol{H_3}$ of Pluronic P123), 2.87(t, 2H, NH_2-C$\boldsymbol{H_2}$-CH_2-O-), 3.1-3.9 (m, -C$\boldsymbol{H_2}$-C$\boldsymbol{H_2}$-O- and -C$\boldsymbol{H_2}$-C$\boldsymbol{H}$(CH_3)-) of PEG and PPG in Pluronic P123).

S2. Synthesis of acid-degradable β-CD PRX

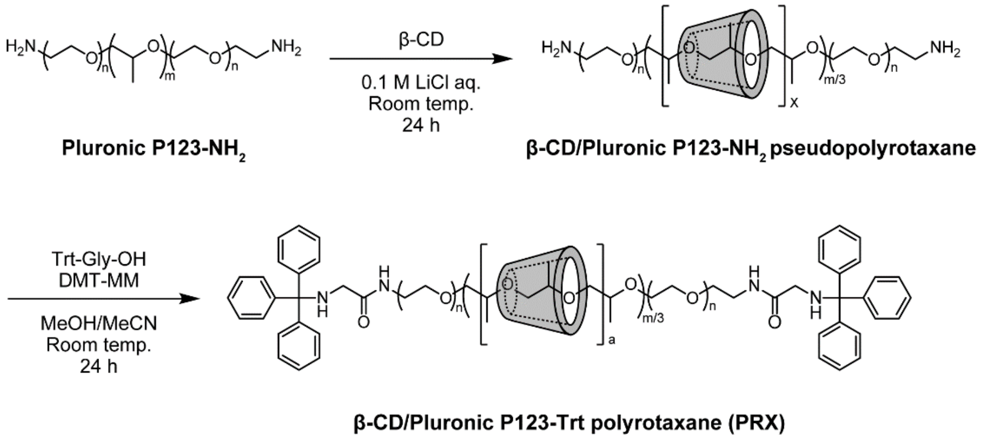

Scheme S2. Synthesis scheme for acid-degradable β-CD/Pluronic P123 polyrotaxane (β-CD PRX), where *n*, *m*, *x*, and *a* denote the number of monomer units in PEG, the number of monomer units in PPG, the number of threaded β-CD in pseudopolyrotaxane, and the number of threaded β-CD in PRX, respectively.

β-Cyclodextrin (β-CD; 40 g, 35.2 mmol; Nihon Shokuhin Kako, Tokyo, Japan) and LiCl (8.48 g, 200 mmol; Tokyo Chemical Industry, Tokyo, Japan) were dissolved in distilled water (2 L) to prepare a β-CD saturated solution. Pluronic P123-NH_2 (4.08 g, 0.73 mmol) dissolved in a small aliquot of water was added to the β-CD saturated solution, and the solution was stirred for 24 h at room temperature. After the reaction, the pseudopolyrotaxane was collected via centrifugation (6,500 rpm, 5 min), and the precipitate was freeze-dried to yield pseudopolyrotaxane (33.35 g).

N-(Triphenylmethyl)glycine (Trt-Gly-OH; 4.55 g, 14.3 mmol; Tokyo Chemical Industry) and 4-(4,6-dimethoxy-1,3,5-triazin-2-yl)-4-methylmorpholinium chloride (DMT-MM; 3.97 g, 14.3 mmol; Fujifilm Wako Pure Chemical) were dissolved in a mixture of methanol (MeOH) and acetonitrile (MeCN) (volume ratio of MeOH:MeCN = 3:10, 264 mL). This solution was added to pseudopolyrotaxane (33.35 g), and the reaction mixture was stirred for 24 h at room temperature. The precipitate was collected via centrifugation (6,500 rpm, 10 min). The raw product was washed with acetone to remove unreacted Trt-Gly-OH and P123-NH_2. The product was dissolved in a small amount of *N*,*N*-dimethyl formamide (DMF; Kanto Chemical), poured into water to reprecipitate the product, and the precipitate was collected via centrifugation (6,500 rpm, 10 min). This process was repeated until the free β-CD and other side products were completely removed. Finally, the collected precipitate was washed 3 times with water to remove DMF. The recovered precipitate was freeze-dried to obtain

polyrotaxane (PRX; 3.85 g, 25.6% yield based on Pluronic P123 mol%). ^{1}H NMR (400 MHz, DMSO-d_6): δ = 1.05 (m, -C***H₃*** of Pluronic P123), 3.1-3.9 (m, -C***H₂***-C***H₂***-O- and -C***H₂***-C***H***(CH₃)- of PEG and PPG in Pluronic P123, H2, H3, H4, H5, and H6 protons of β-CD), 4.44 (m, O6H proton of β-CD), 4.83 (s, H1 proton of β-CD), 5.5-5.9 (m, O2H and O3H protons of β-CD), 7.20 (t, Trt group), 7.29 (t, Trt group), 7.37 (t, Trt group).

S3. Cell culture

DC2.4 cells, a mouse dendritic cell line, were obtained from Merck (Darmstadt, Germany). DC2.4 cells were cultured in RPMI-1640 (Fujifilm Wako Pure Chemical) supplemented with 4 mM L-glutamine, 25 mM 2-[4-(2-hydroxyethyl)-1-piperazinyl]ethanesulfonic acid (HEPES), 10% FBS, non-essential amino acids (Fujifilm Wako Pure Chemical), 50 μM 2-mercaptoethanol (Fujifilm Wako Pure Chemical), 100 units/mL penicillin, and 100 μg/mL streptomycin (Fujifilm Wako Pure Chemical) in 5% CO_2 at 37 °C.

S4. Synthesis of fluorescently labeled carboxylated polyrotaxanes

The fluorescently labeled carboxylated β-CD PRXs were prepared according to a previously described method with slight modification [S1]. Briefly, CMC-PRX (50 mg, 2.11 μmol of CMC-PRX), 4,4-difluoro-5,7-dimethyl-4-bora-3a,4a-diaza-*s*-indacene-3-propionyl ethylenediamine hydrochloride (BODIPY FL EDA; Thermo Fisher Scientific, Waltham, MA, USA; 790 μg, 2.13 μmol), and DMT-MM (2.95 mg, 10.7 μmol) were dissolved in carbonate buffer (10 mM $NaHCO_3$/Na_2CO_3 at pH 9.2; 5 mL), and the solution was stirred for 24 h at room temperature. After the reaction, the resulting polymer was purified via dialysis against water for 3 days using Spectra/Por 1 (molecular weight cut-off of 6,000–8,000). The recovered solution was freeze-dried to yield BODIPY-labeled CME-PRX (BODIPY-CMC-PRX; 31 mg, 62% yield). BODIPY-labeled CEC-PRX and CPC-PRX were synthesized and purified in the same manner.

The fluorescently labeled HEE-PRX was prepared according to a previously described method with slight modification [S2]. Briefly, HEE-PRX (50 mg, 1.84 μmol of HEE-PRX) and CDI (3.0 mg, 18.5 μmol) were dissolved in dehydrated DMSO (2.5 mL) under a nitrogen atmosphere and the solution was stirred for 24 h at room temperature. BODIPY FL EDA (686 mg, 1.85 μmol) was added to the reaction mixture, and the solution was stirred for 24 h at room temperature with protection from light. After the reaction, the resulting polymer was purified via dialysis against water for 3 days using Spectra/Por 1 (molecular weight cut-off of 6,000–8,000). The recovered solution was freeze-dried to yield BODIPY-labeled HEE-PRX (BODIPY-HEE-PRX; 37 mg, 74% yield).

The number of BODIPY molecules modified on the carboxylated PRXs and HEE-PRX was

determined by measuring the absorbance at 503 nm using a V-550 UV/VIS spectrophotometer (Jasco, Tokyo, Japan). In cell experiments, unlabeled and BODIPY-labeled PRXs were mixed to adjust the fluorescence intensities of BODIPY-labeled carboxylated PRXs and BODIPY-HEE-PRX.

S5. References

[1] Matsui H, Tamura A, Osawa M, et al. Scavenger receptor A-mediated targeting of carboxylated polyrotaxanes to macrophages and the impacts of supramolecular structure. Macromol Biosci. 2018;18:1800059.

[2] Shibaguchi K, Tamura A, Terauchi M, et al. Mannosylated polyrotaxanes for increasing cellular uptake efficiency in macrophages through receptor-mediated endocytosis. Molecules. 2019;24:439.

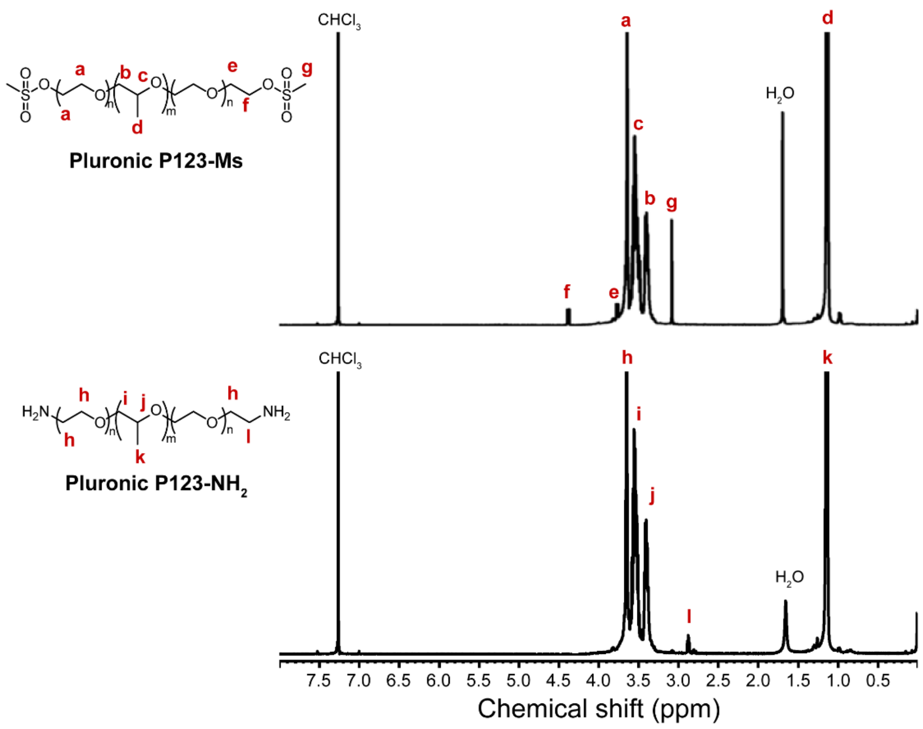

Figure S1. ^{1}H NMR spectra of mesyl-terminated Pluronic P123 and amine-terminated Pluronic P123 in CDCl3 at 25 °C.

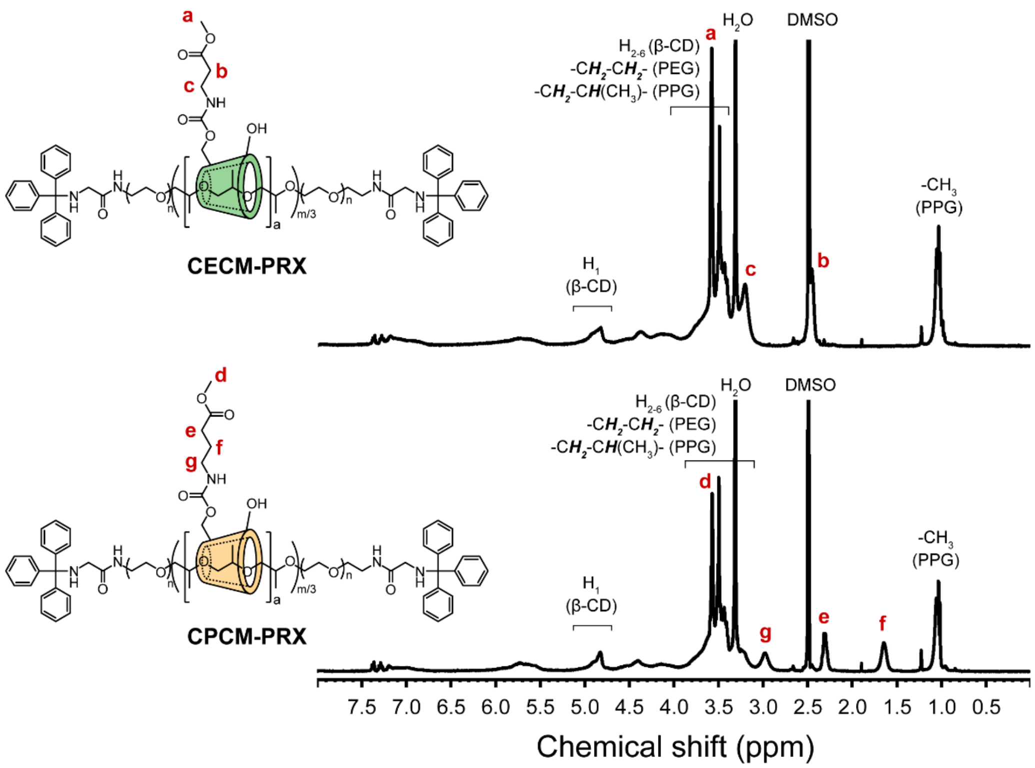

Figure S2. ^{1}H NMR spectra of CECM-PRX and CPCM in DMSO-d_6 at 25 °C.

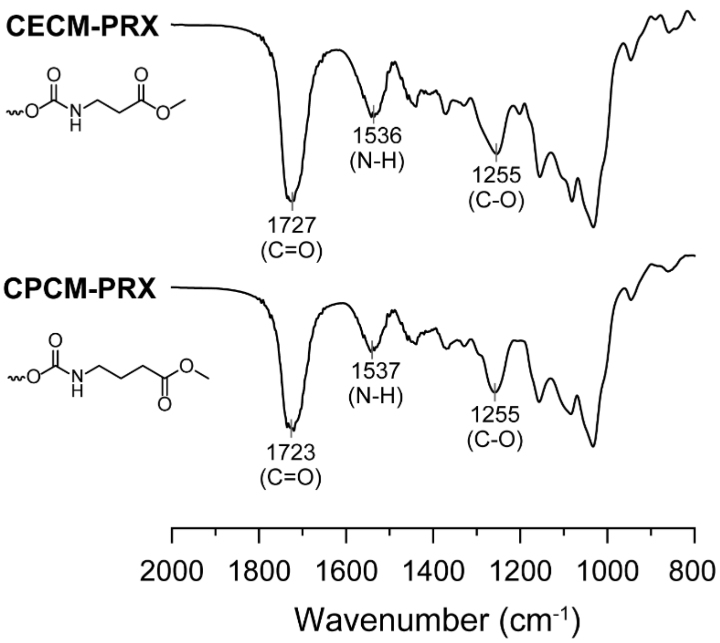

Figure 3. FT-IR spectra of CECM-PRX and CPCM-PRX.

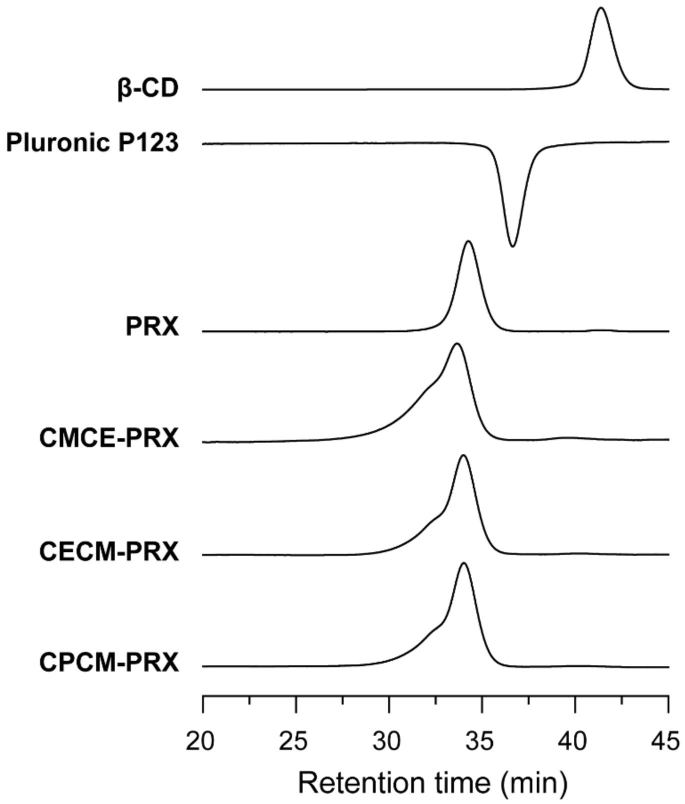

Figure S4. SEC charts of β-CD, Pluronic P123, PRX, CMCE-PRX, CECM-PRX, and CPCM-PRX in DMSO at 60 °C.

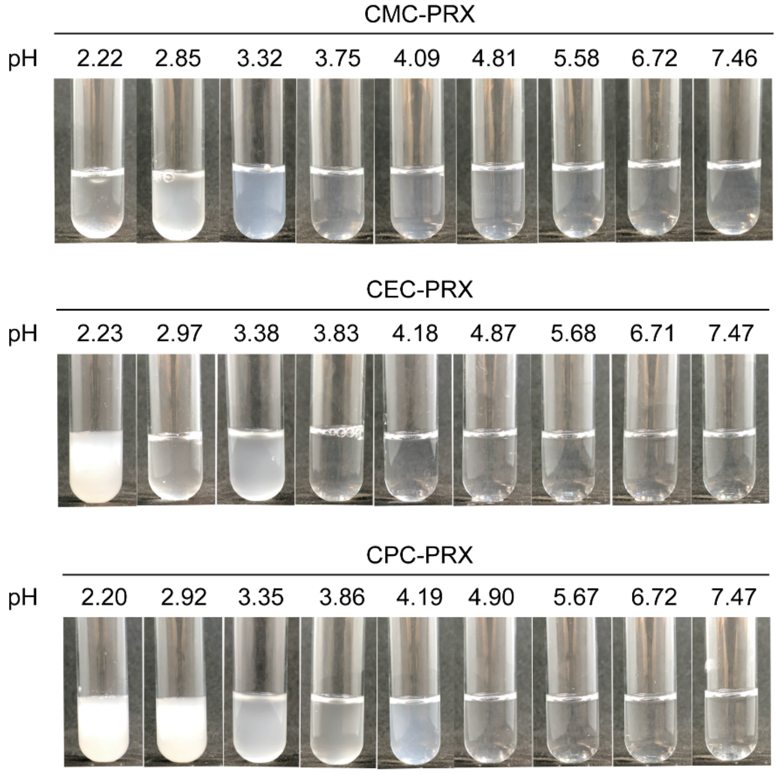

Figure S5. Images of the aqueous solutions of carboxylated PRXs at various pH conditions (5.0 mg/mL).

Table S1. Characterization of BODIPY-labeled PRXs.

Code	Number of threaded β-CDs	Number of functional groups on PRX (per β-CD)		Number of BODIPY on PRX[a]
		Carboxy	HEE	
BODIPY-CMC-PRX	12.5	31.3 (2.5)	-	0.108
BODIPY-CEC-PRX	12.5	27.5 (2.2)	-	0.104
BODIPY-CPC-PRX	12.5	22.5 (1.8)	-	0.101
BODIPY-HEE-PRX	12.5	-	50.0 (4.0)	0.229

[a]Determined using UV-vis absorption.

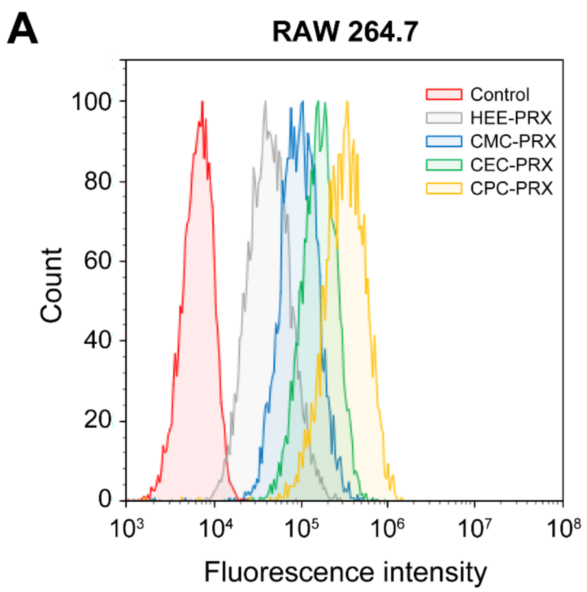

Figure S6. Fluorescence intensity histograms of RAW 264.7 (A) and NIH/3T3 cells (B) treated with BODIPY-labeled carboxylated PRX (200 µM threaded β-CD; CMC-PRX = 387 µg/mL, CEC-PRX = 386 µg/mL, CPC-PRX = 381 µg/mL) and HEE-PRX (200 µM threaded β-CD; 432 µg/mL) for 24 h.

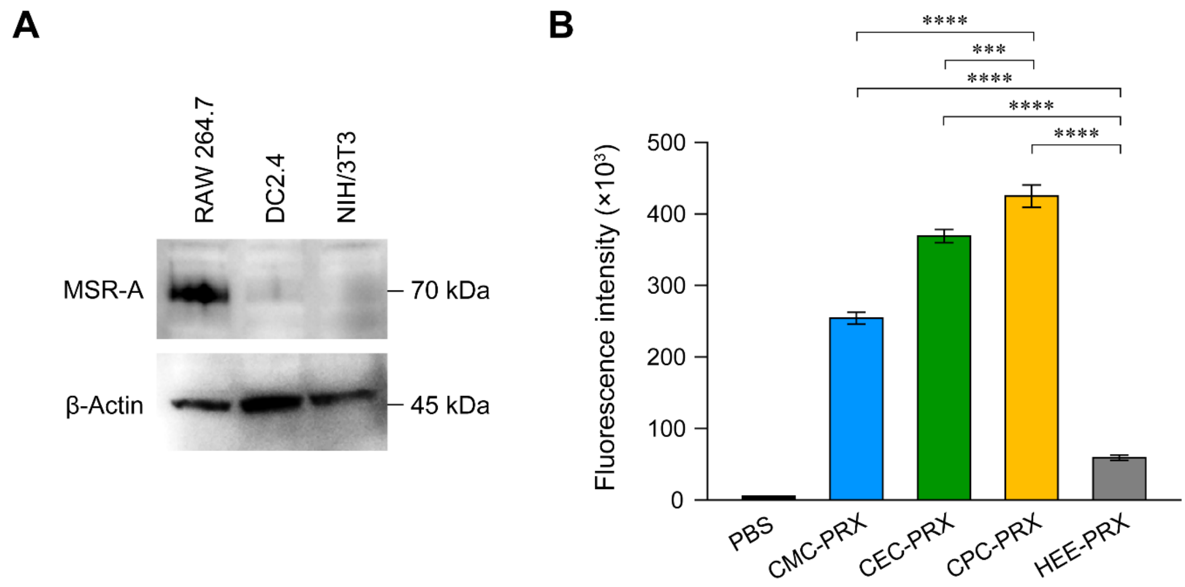

Figure S7. (A) Immunoblot analysis for MSR-A and β-actin expression in RAW 264.7, DC 2.4, and NIH/3T3 cells. (B) Fluorescence intensity of DC 2.4 cells treated with BODIPY-labeled PRXs for 24 h (n = 3, ***$P < 0.001$, ****$P < 0.0001$).
